# piR-37524 Overexpression in Colorectal Cancer: A Potential Diagnostic Bio-Marker and Therapeutic Target

**DOI:** 10.32604/or.2026.074981

**Published:** 2026-03-23

**Authors:** Jiaxi Li, Deepak Iyer, Siming Sui, Zheng Huang, Ryan Wai-Yan Sin, Abraham Tak-Ka Man, Wai-Lun Law, Chi-Chung Foo, Lui Ng

**Affiliations:** Department of Surgery, School of Clinical Medicine, Li Ka Shing Faculty of Medicine, The University of Hong Kong, Hong Kong SAR, 999077, China

**Keywords:** Colorectal cancer, piRNAs, tumor biomarker

## Abstract

**Objectives:**

Piwi-associated RNAs are small non-coding RNAs implicated in cancer, yet few have been characterized in colorectal cancer (CRC). This study aimed to identify a CRC-related piRNA and investigate its clinical relevance, biological function, and biomarker potential.

**Methods:**

Candidates were identified by reanalysis of small-RNA sequencing. piR-37524 was quantified by quantitative real-time polymerase chain reaction (qRT-PCR) in colorectal cancer tissues, matched adjacent non-tumor tissues, colorectal adenomas, liver metastases, and serum samples from patients and healthy controls. Clinicopathological correlations and diagnostic performance were evaluated. Functional assays included 3-(4,5-dimethylthiazol-2-yl)-2,5-diphenyltetrazolium bromide (MTT) proliferation, colony formation, and wound-healing migration in HCT116 and HT29 cells after piR-37524 inhibition. RNA sequencing and Western blotting examined epithelial–mesenchymal transition (EMT) markers and nuclear factor-kappa B (NF-κB) components.

**Results:**

piR-37524 was significantly overexpressed in CRC compared with adjacent non-tumor tissues and was associated with larger tumor size, poorer differentiation, and distant metastasis. Elevated expression was also observed in colorectal adenomas and liver metastases. Serum piR-37524 levels were increased in patients with adenomas and CRC compared with healthy controls, indicating diagnostic potential. Functional assays demonstrated that piR-37524 inhibition suppressed CRC cell proliferation and migration, accompanied by changes consistent with epithelial–mesenchymal transition regulation. Mechanistic analyses implicated tumor necrosis factor alpha-induced protein 3 (TNFAIP3)-associated NF-κB signaling.

**Conclusions:**

piR-37524 is an oncogenic piRNA in CRC that promotes progression via the TNFAIP3/NF-κB/EMT axis, serving as a potential pan-stage diagnostic biomarker and therapeutic target.

## 1 Introduction

Colorectal cancer (CRC) ranks as a major global health concern, accounting for 9.6% of incident malignancies and 9.3% of cancer-related mortalities worldwide [1]. Despite decades of research elucidating its pathogenesis and molecular mechanisms, the rising global burden of CRC underscores persistent gaps in our understanding of disease progression. This necessitates the identification of novel biomarkers and therapeutic targets to improve clinical management strategies.

In recent years, non-coding RNAs (ncRNAs) have been increasingly acknowledged as crucial modulators of tumor development. Among these, microRNAs (miRNAs) have been extensively shown to modulate cancer-related pathways [2,3], while long non-coding RNAs (lncRNAs) have emerged as important drivers in tumor initiation and progression [4–6]. Recently, Piwi-interacting RNAs (piRNAs)—a class of 26–31 nucleotide small ncRNAs initially characterized in germline cells for their roles in transposon silencing, genome stability, and stem cell maintenance. Several studies have, however, demonstrated that piRNAs can also regulate the somatic tissues and influence the development or progression of several cancers [7–9]. In CRC, several piRNAs have been implicated in oncogenesis, including piR-004530 [10], piR-1245 [11], piR-54265 [12], piR-18849 [13], and piR-57125 [14], while piR-5937 and piR-28876 exhibit downregulation in tumor tissues compared to the adjacent normal tissues [15]. Indeed, there are several more research groups actively working on the discovery and characterization of novel piRNAs that are associated with the CRC carcinogenesis process [16], thereby improving our understanding of the overall regulatory biology of these molecules. With properties like miRNAs, such as small size, high stability in multiple body tissues and fluids, yet with a high degree of diversity, piRNAs are perfect candidates for functioning as predictive biomarkers, druggable targets, or even therapeutic agents.

Building on our previous profiling of the CRC piRNAome [8], reanalysis of small RNA sequencing highlighted piR-37524 as overexpressed in colorectal cancer relative to adjacent non-malignant tissue. This direction of change was corroborated in preliminary validation using an independent assay. To our knowledge, piR-37524 has not been systematically investigated in colorectal cancer.

Building on our previous profiling of the CRC piRNAome, this study aimed to characterize the clinical and biological significance of piR-37524 in CRC. We sought to determine its expression pattern in colorectal tumors, precursor adenomas, and distant metastases, and to evaluate whether tissue and serum levels of piR-37524 are associated with clinicopathological features and patient outcomes. We further aimed to investigate the effects of piR-37524 modulation on CRC cell proliferation and migration and to explore the underlying molecular mechanisms. We hypothesized that piR-37524 functions as an oncogenic piRNA that promotes colorectal cancer progression and that its expression may serve as a diagnostic and prognostic biomarker.

## 2 Material and Methods

### 2.1 Patient Tissue and Blood Samples

This study adhered to the principles of the Declaration of Helsinki and was approved by the Institutional Review Board/Ethics Committee of The University of Hong Kong (UW 21-114; approval date 2 August 2021). Written informed consent was obtained from all participants prior to enrolment.

Fresh surgically resected colorectal tumor tissues and matched adjacent normal mucosa were collected from 64 patients undergoing surgery at the Department of Surgery, Queen Mary Hospital, The University of Hong Kong. The series comprised normal colon (N = 64), colorectal adenoma (CoAd) (N = 13), non-metastatic CRC M0 (N = 47), metastatic CRC M1 (N = 17), and liver metastasis (N = 9). Immediately after resection, tissue specimens were snap-frozen in liquid nitrogen and stored at −80°C until analysis. In addition, serum samples were obtained from 26 patients with colorectal adenoma (prior to colonoscopy), 38 patients with CRC (pre-operative), and 36 healthy control individuals without a history of CRC or adenomatous polyps. Whole blood was centrifuged at 400× *g* for 10 min, and the clarified serum was aliquoted into sterile 2 mL tubes and stored at −80°C until analysis. Clinicopathological variables were retrieved from the patients’ medical records.

### 2.2 Cell Culture and Transfection

HCT116 (CCL-247) and HT29 (HTB-38) CRC cell lines were purchased from the American Type Culture Collection (ATCC, Manassas, VA, USA). Cell line authentication was performed by Short Tandem Repeat (STR) profile analysis at Centre for PanorOmic Sciences (Hong Kong, China). No mycoplasma contamination was detected throughout the experiments. All cells used in these experiments were cultured in Dulbecco’s Modified Eagle Medium (DMEM) (Gibco, Cat. No. 11965-092, Grand Island, NY, USA) supplemented with 10% fetal bovine serum (FBS) (Gibco, Cat. No. 10099-141) and 1% penicillin–streptomycin (Gibco, Cat. No. 15140-122). Cells were kept at 37°C in a humidified incubator containing 5% CO_2_. For transfection, 2 × 10^5^ cells were seeded into each well of a 12-well plate and transfected with piR-37524 inhibitors or negative control (NC) oligonucleotides using Lipofectamine 3000 (Invitrogen, Cat. No. L3000008, Waltham, MA, USA) according to the manufacturer’s protocol. The piR-37524 inhibitors and NC oligonucleotides were designed and synthesized by RiboBio (Guangzhou, China).

### 2.3 RNA Extraction, cDNA Synthesis, and Quantitative PCR

Total RNA was extracted from tissue specimens and serum samples with the mirVana™ miRNA Isolation Kit (Thermo Fisher Scientific, Cat. No. AM1560, Lenexa, KS, USA). All procedures were carried out in accordance with the manufacturer’s protocol. cDNA synthesis and quantitative real-time PCR were performed as previously described, with minor modifications [8]. Primer sequences used are listed in Table S1. For tissue specimens, piRNA expression levels were normalised to RNU6B, whereas serum piRNA levels were normalised to miR-92a-3p. Relative expression was expressed as ΔCt values.

### 2.4 MTT Assay

Cell proliferation was quantified using an MTT assay (Invitrogen, Cat. No. M6494). After transfection, 1 × 10^3^ cells were seeded per well in 96-well plates and cultured for 24, 48, 72, and 96 h. 10 μL MTT solution (5 mg/mL) was added to each well, and the plates were incubated for a further 3 h. The resulting formazan crystals were dissolved in DMSO (Synth, Cat. No. D2650, Diadema, SP, Brazil), and absorbance at 570 nm (with 630 nm as reference wavelength for background subtraction) was measured using a Multiskan™ FC microplate reader (Thermo Fisher Scientific, Model 51119000).

### 2.5 Colony Formation Assay

Transfected cells were seeded in 6-well plates at a density of 1 × 10³ cells per well and cultured for 10–14 days until visible colonies formed. Colonies were then washed twice with PBS, fixed with 100% ice-cold methanol for 30 min, and stained with 0.2% crystal violet (Sigma-Aldrich, Cat. No. C0775, St. Louis, MO, USA) for 15 min at room temperature. Colony numbers were counted under a light microscope (Olympus, Model CKX53, Tokyo, Japan).

### 2.6 Wound Healing Assay

Cell migration was evaluated using a wound-healing assay. Transfected cells (2 × 10^5^/well) were seeded in 12-well plates and grown to a confluent monolayer. A linear scratch was introduced into the monolayer using a 10 µL sterile pipette tip, and detached cells were removed by washing with PBS. Fresh serum-free culture medium (DMEM, Gibco, Cat. No. 11965-092) was added, and images were captured at 0, 48, and 72 h using an Olympus CKX53 microscope (Evident Corporation, Model CKX53, Tokyo, Japan). Wound closure was quantified using ImageJ software (Version 1.54f, National Institutes of Health, Bethesda, MD, USA).

### 2.7 RNA Sequencing and Analysis

Total RNA was isolated from transfected cells using TRIzol reagent (Invitrogen, Cat. No. 15596026) following the manufacturer’s protocol. RNA integrity was assessed with an Agilent 2100 Bioanalyzer (Agilent Technologies, Model G2939BA, Santa Clara, CA, USA), and only samples with an RNA integrity number (RIN) > 7 were used for library preparation. Libraries were generated using the TruSeq RNA Sample Preparation Kit (Illumina, Cat. No. RS-122-2001, San Diego, CA, USA) and sequenced on an Illumina HiSeq 2500 platform. Sequencing reads were quality-filtered, and high-quality reads were aligned to the human reference genome (GRCh38) using HISAT2. Differential expression analysis was carried out with the DESeq2 R package (Version 1.38.3), applying a threshold of *p* < 0.05 and absolute log_2_ fold change (FC) > 1. Differentially expressed genes (DEGs) were visualised in a volcano plot generated with ggplot2 in R. Overlaps among DEG sets were summarised using Venn diagrams generated with the online tool at https://bioinformatics.psb.ugent.be/webtools/Venn/.

### 2.8 Prediction of piRNA Target Genes

Potential target genes of piR-37524 were predicted using miRanda software (http://www.bioinformatics.com.cn). Candidate interactions between piR-37524 and mRNA 3^′^-UTRs were selected based on sequence complementarity and binding energy criteria.

### 2.9 Western Blot Analysis

20 µg total protein lysates were separated by 10% SDS–polyacrylamide gel electrophoresis at 120 V for 90 min and electrotransferred onto PVDF membranes (Bio-Rad, Cat. No. 1620177, CA, USA). Membranes were blocked in TBST containing 5% BSA (Sigma-Aldrich, Cat. No. A215) for 1 h at room temperature. Primary antibodies were used to incubate overnight at 4°C against E-cadherin (1:1000, Cell Signaling Technology, Cat. No. 3195, Danvers, MA, USA), β-catenin (1:1000, Cell Signaling Technology, Cat. No. 8480), Snail (1:1000, Cell Signaling Technology, Cat. No. 3879), TNFAIP3 (1:1000, ABclonal, Cat. No. A1741, Woburn, USA), and GAPDH (1:5000, Santa Cruz Biotechnology, Cat. No. sc-47724, Dallas, TX, USA). After three washes in TBST, membranes were incubated with horseradish peroxidase-conjugated secondary antibodies (1:10000, Invitrogen, Cat. No. 31460) for 1 h at room temperature. Protein bands were detected using enhanced chemiluminescence (ECL) reagents (Thermo Fisher Scientific, Cat. No. 32106).

### 2.10 Statistical Analysis

For tissue and serum piRNA measurements, relative expression was expressed as −ΔCt values. The distribution of the data was assessed using the Kolmogorov–Smirnov or Shapiro–Wilk test. Differences between two groups were analysed using Student’s *t* test or the Mann–Whitney U test, as appropriate, and comparisons among more than two groups were evaluated using one-way ANOVA or the Kruskal–Wallis test. Receiver operating characteristic (ROC) curves were constructed to evaluate the diagnostic performance of serum piR-37524. A *p*-value < 0.05 was considered statistically significant. All statistical analyses were performed using GraphPad Prism version 8.0.1 (GraphPad Inc., San Diego, CA, USA).

## 3 Results

### 3.1 piR-37524 Is Overexpressed in CRC and Correlates with Critical Clinical Features Associated with Tumor Development and Progression

**Figure 1:**
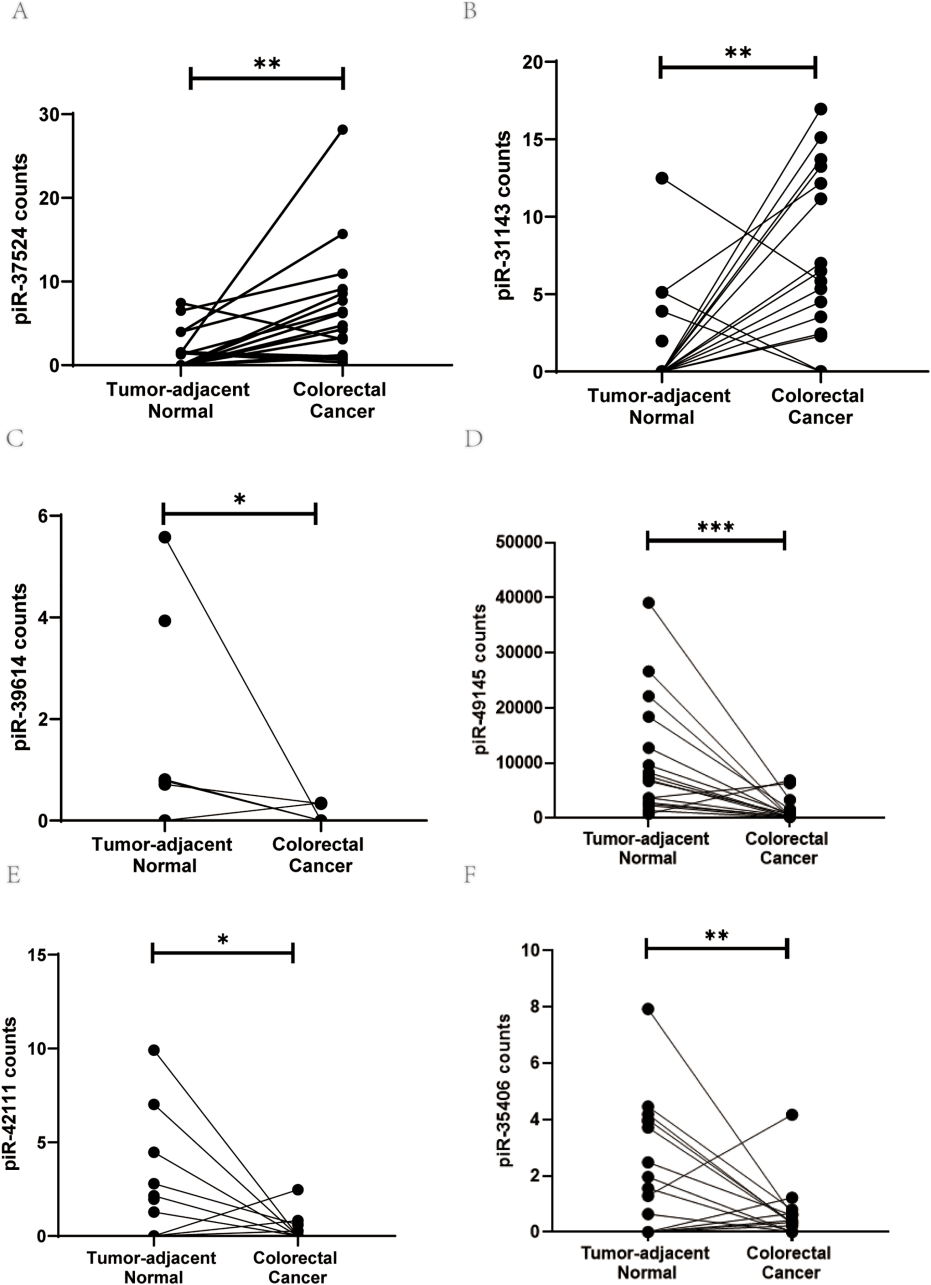
PiRNA discovery from the next-generation sequencing dataset. Expression levels (counts) of (**A**) piR-37524, (**B**) piR-31143, (**C**) piR-39614, (**D**) piR-49145, (**E**) piR-42111, and (**F**) piR-35406 in CRC tumor tissues compared with non-malignant adjacent colorectal tissue. *p < 0.05, **p < 0.01, ***p < 0.001. n = 3

To discover unexplored transcriptional addiction in CRC, we surveyed our previously published small RNA sequencing dataset generated from 18 CRC patients (18 CRC specimens and 17 tumor-adjacent non-cancerous tissues) [8] and identified 6 piRNAs (piR- 37524, piR-31143, piR-39614, piR-49145, piR-42111, and piR-35406) showing differential expression (log_2_-fold change ±2) between CRC tissues and adjacent normal tissues (Fig. 1A–F). These piRNAs were selected based on their differential expression and relevance in cancer, as supported by existing literature (Table S2).

**Figure 2:**
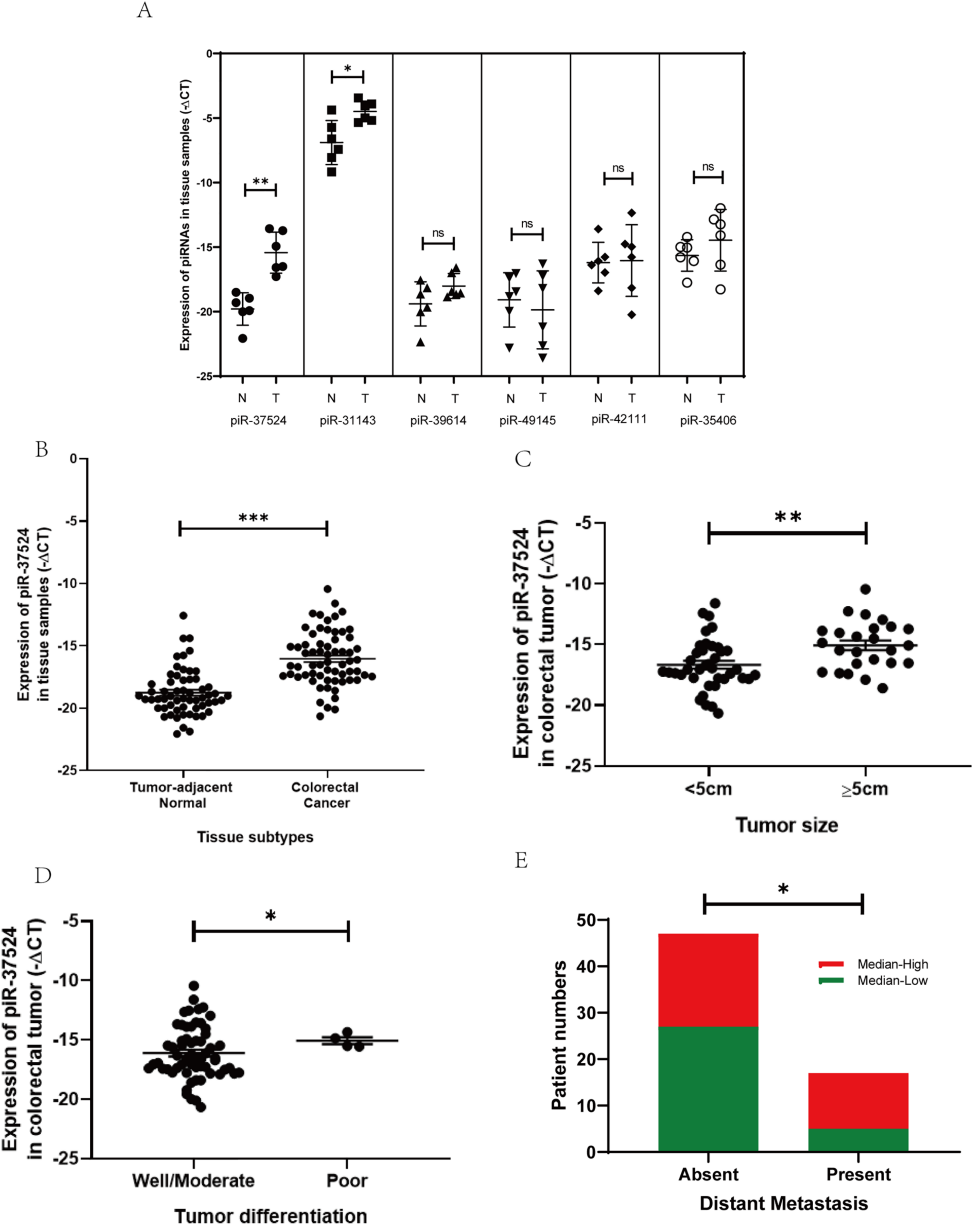
Expression of piR-37524 in colorectal cancer (CRC) tissues and tumor-adjacent non-malignant tissue. (**A**) The 6 paired samples (CRC tumor vs. adjacent normal) form a preliminary validation cohort to screen candidate piRNAs. (**B**) Quantitative polymerase chain reaction (PCR) analysis of piR-37524 expression in CRC tissues and matched normal samples, and its association with (**C**) primary tumor size, (**D**) histological differentiation, and (**E**) distant metastasis in 64 CRC patients enrolled in the validation cohort. *p < 0.05, **p < 0.01, ***p < 0.001 ns: not significant. n = 3

To validate our results from the next-generation sequencing dataset, we performed a preliminary profiling of the expression of these piRNAs in a small patient group comprising 6 primary CRC tissues and the corresponding adjacent non-malignant tissues. The expression of piRNAs within the 6 paired tumor and non-tumor resection specimens was normalized against the gene expression of RNU6B, and the data were represented (as−ΔCt (negative delta Ct). Only piR-37524 and piR-31143 demonstrated a statistically significant change in expression within the CRC tissues relative to the adjacent normal (piR-37524: *p* = 0.0081; piR-31143: *p* = 0.0186) (Fig. 2A). While piR-31143 had been previously explored in CRC (16), we focused on the novel piRNA, piR-37524.

To further expand our pilot results from the preliminary profiling, we performed a validation of the expression of piR-37524 using real-time PCR in a larger patient group comprising 64 primary CRC tissues and the corresponding adjacent normal resection specimens. The patient cohort had a median age of 67 years, with primary tumors located in the colon (71.8%), and rectum (including rectosigmoid region) (28.1%) (Table 1). Most patients were male (57.8%), and tumors were classified according to AJCC guidelines as stage I (4.6%), II (28.1%), III (40.6%), or IV (26.6%) (Table 1).

**Table 1:** Correlation between the expression of piR-37524 and the clinical features of 64 CRC patients recruited for the validation phase

Characteristics	piR-37524 expression		*p*-Value
	Low, *n* = 32	High, *n* = 32	
**Age(years)**			
<60	9	11	
≥60	23	21	0.4111
Gender			
Male	16	21	
Female	16	11	0.3350
Tumor location			
Colon	23	23	
Rectum (including rectosigmoid)	9	9	0.8967
Tumor size			
<50 mm	24	15	0.0031*
≥50 mm	8	17	
Tumor differentiation			
Well/Moderate	32	28	0.0264*
Poor	0	4	
T classification			
T1–T2	4	3	0.6083
T3	22	20	
T4	6	9	
Lymph node metastasis			
N0	11	14	0.8750
N1	10	8	
N2	11	10	
Distant metastasis			
No	27	20	0.0476*
Yes	5	12	
TNM stage			
I	2	1	0.2045
II	8	10	
III	17	9	
IV	5	12	

Note: *p < 0.05.

Our data demonstrated that piR-37524 was significantly overexpressed, by over 30-fold in primary CRC tissues relative to the adjacent normal tissues (median: −16.41 vs. −19.18; *p* < 0.001) (Fig. 2B), with 71.8% of patients showing a T/N >2-fold change in the expression of the piRNA. We subsequently performed a clinicopathological analysis to identify the correlation of the expression of piR-37524 with the overall development and progression of CRC within the patient cohort. Statistical analysis using unpaired Student’s *t*-test demonstrated that the expression of piR-37524 positively correlated with the primary tumor size (Fig. 2C). Specifically, 68% of patients with tumors ≥ 5 cm (39% of total) showed median-high piR-37524 expression, compared to 38.5% of patients with smaller tumors (<5 cm) (*p* = 0.0031) (Table 1). Poorly differentiated tumors also showed higher piR-37524 expression than well to moderately differentiated tumors (median: −15.19 vs. −16.55, *p* = 0.0264) (Fig. 2D). Expression of piR-37524 was also found to reflect the metastatic potential of the primary CRC tumors. About 70% of the patients exhibiting distant metastases showed a median-high expression of piR-37524, while only 42.5% of the non-metastatic patients expressed a median-high level of the piRNA (*p* = 0.0476) (Fig. 2E). Other clinicopathological factors did not correlate with piR-37524 expression.

### 3.2 High Expression of piR-37524 Is Observed in CoAd and Distant Metastases

**Figure 3:**
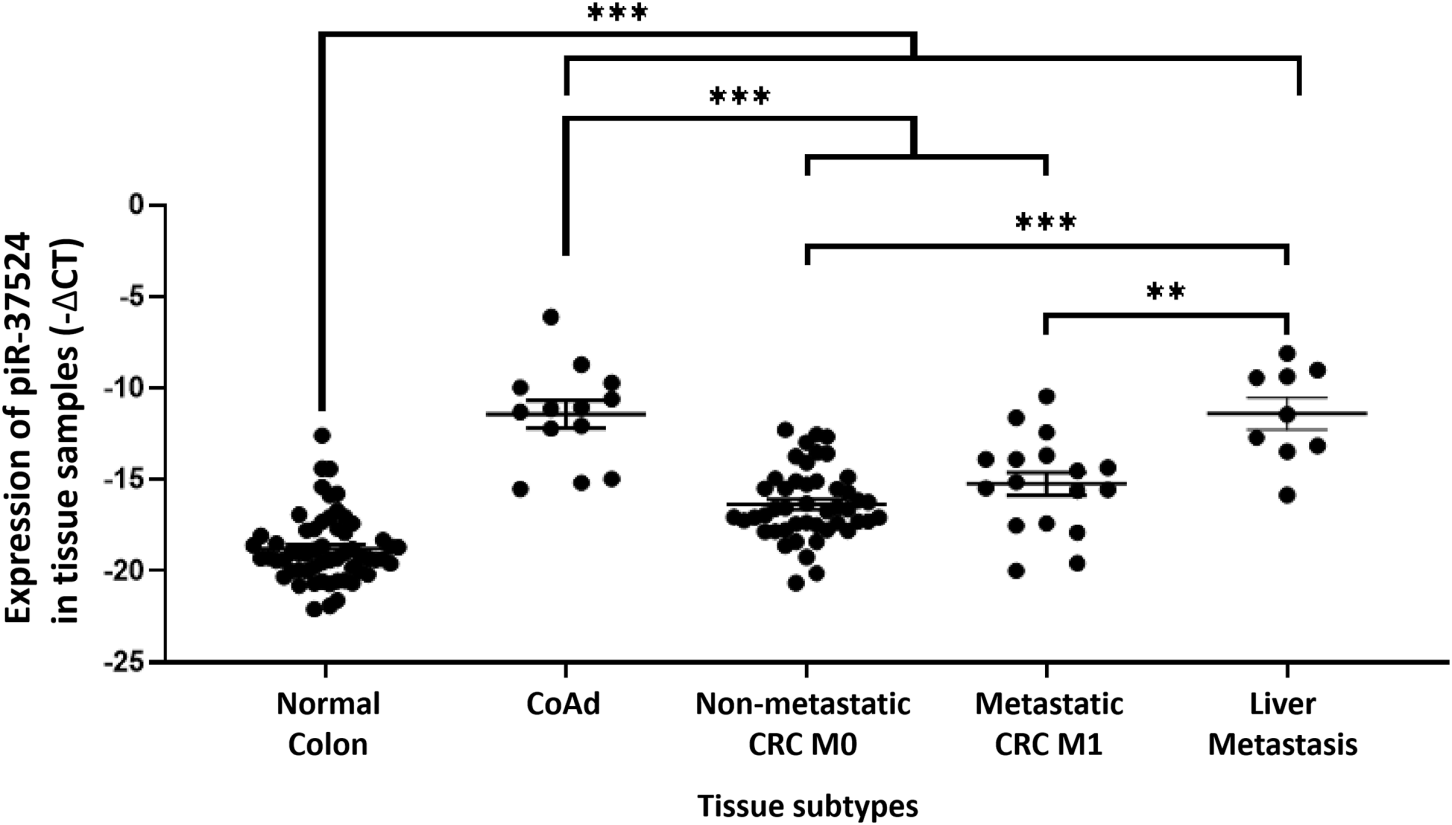
Expression of piR-37524 in normal colon and multiple developmental stages of CRC. Quantitative PCR-based expression analysis of piR-37524 in normal colon (N = 64), CoAd (N = 13), non-metastatic CRC M0 (N = 47), metastatic CRC M1 (N = 17), and liver metastasis (N = 9). **p < 0.01, ***p < 0.001. n = 3

Since the expression of piR-37524 correlated clinically with tumor size, differentiation, and metastasis, it was imperative to investigate this piRNA across multiple developmental stages of CRC to get a better understanding of the distinct roles of piR-37524 in the disease. Consequently, we profiled the expression of piR-37524 within CoAd (N = 13), non-metastatic CRC M0 (N = 47), metastatic CRC M1 (N = 17), as well as distant liver metastases (N = 9). Within the study population, while CoAd and liver metastases represented the preliminary stage or risk factor for CRC or new cancer (at a distant site) development, metastatic tumors formed the late stage of CRC progression. Our results demonstrated that overall, the expression of piR-37524 was significantly higher in CoAd, CRC, and liver metastases than in adjacent non-tumor tissue (N = 64) (*p* < 0.001) (Fig. 3). Notably, a significantly higher expression of the piRNA was observed within the individuals with adenomatous polyps relative to the non-metastatic CRC (median: −11.12 vs.−16.61, *p* < 0.001) and metastatic CRC (median: −11.12 vs. −15.13, *p* = 0.0006) (Fig. 3). Liver metastases also showed higher piR-37524 expression than the M0 (median: −11.43 vs. −16.61, *p* = 0.0003) and the M1 (median: −11.38 vs. −15.13, *p* = 0.0024) CRC groups (Fig. 3). However, there was no significant difference in piR-37524 expression between CoAd and liver metastases (median: −11.12 vs. −11.38, *p* = 0.9783).

### 3.3 PiR-37524 Is a Potential Blood-Based Disease Screening Biomarker in CRC

Given the high piR-37524 expression in early CRC development and distant metastasis, we evaluated its potential as a screening biomarker. We measured serum piR-37524 expression in 64 patients with CoAd (N = 26) or CRC (N = 38) and in 36 healthy controls. Expression was normalized to miR-92a-3p (−ΔCt).

**Figure 4:**
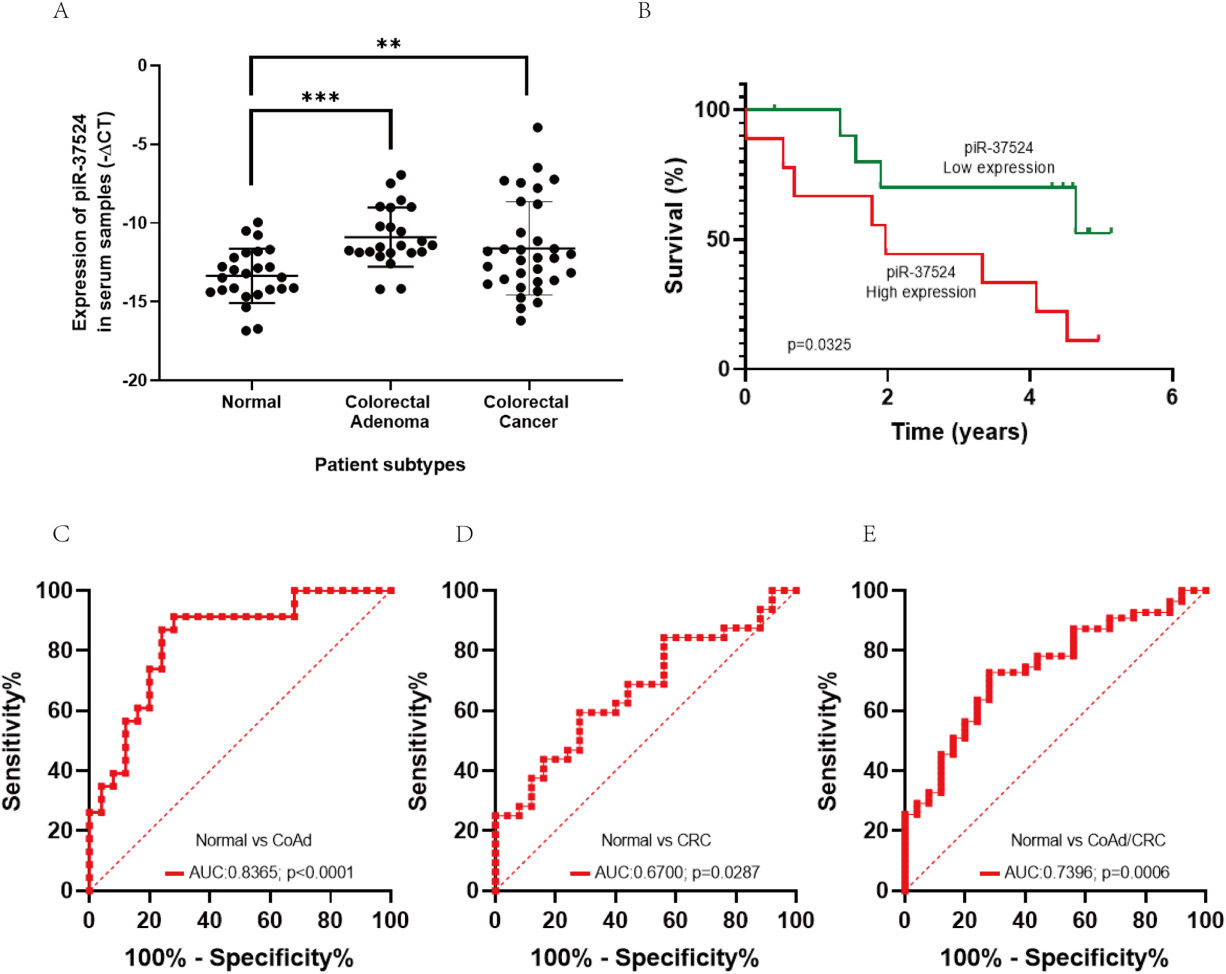
Role of piR-37524 as a diagnostic serum biomarker in CRC. (**A**) Quantitative PCR-based expression analysis of piR-37524 in serum samples from non-cancer, CoAd, and CRC patients. (**B**) Kaplan Meir survival curves showing the overall survival of CRC patients with a median-high or a median-low piR-37524 expression. A standard receiver operating (ROC) curve was plotted to assess the diagnostic performance of piR-37524 in serum samples from (**C**) normal versus CoAd patients, (**D**) normal versus CRC patients, and (**E**) normal versus CoAd and CRC patients. **p < 0.01, ***p < 0.001. n = 3

Serum piR-37524 expression was significantly higher in patients with CoAd (median: −11.44 vs. −13.44, *p* < 0.001) and CRC (median: −12.22 vs. −13.44, *p* = 0.0078) compared to healthy controls (Fig. 4A). There was no significant difference between CoAd and CRC serum levels. High serum piR-37524 expression correlated with poor overall survival in CRC patients (*p* = 0.0325) (Fig. 4B).

We subsequently plotted receiver operating characteristic (ROC) curves to have a better understanding of the diagnostic accuracy of piR-37524 within serum samples. As shown in Fig. 4C, piR-37524 significantly differentiates between the CoAd patients and normals with an AUC of 0.8365 (95% CI: 0.7215–0.9515, Sensitivity: 86.9%, Specificity: 76%, *p* < 0.0001). However, for the CRC vs. normal group, we obtained a lower AUC of 0.6700 (95% CI: 0.5304–0.8096, Sensitivity: 62.5%, Specificity: 56%, *p* = 0.0287) (Fig. 4D). Although by combining the CoAd and the CRC patients in a single group, we managed to enhance the AUC slightly to 0.7396 (95% CI: 0.6270–0.8522, Sensitivity: 72.73%, Specificity: 72%, *p* = 0.0006), thus improving the detection accuracy of CRC patients and individuals at risk of CRC (CoAd) from normal (Fig. 4E).

### 3.4 piR-37524 Modulates the Proliferation and Migration of Colorectal Cancer Cells

**Figure 5:**
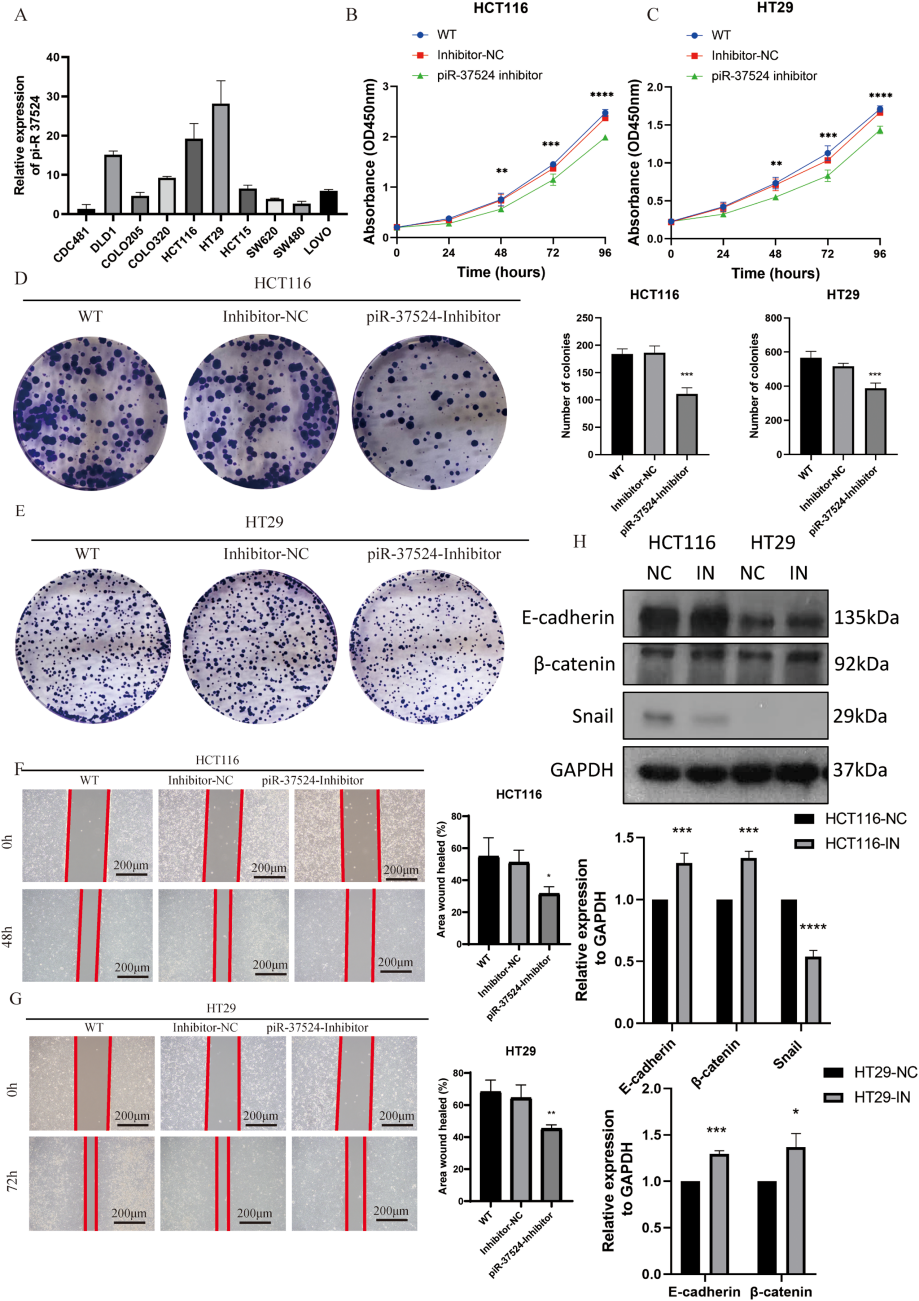
piR-37524 modulates the proliferation and migration of colorectal cancer cells. (**A**) Expression levels of piR-37524 in different colorectal cancer cell lines as determined by qRT-PCR. (**B**,**C**) Proliferation of HCT116 and HT29 cells transfected with piR-37524 inhibitors or a negative control as measured by MTT assay at 24, 48, 72, and 96 h. (**D**,**E**) Colony formation assay of HCT116 and HT29 cells transfected with piR-37524 inhibitors or a negative control. Representative images of colonies and quantification of colony numbers are shown. (**F**,**G**) Wound healing assay to assess the migration of HCT116 and HT29 cells transfected with piR-37524 inhibitors or a negative control. Representative images of wound closure at 0, 48, or 72 h and quantification of wound healing are shown. (**H**) WB showed decreased Snail protein expression in the piR-37524-inhibited HCT116 cell line, as well as increased protein levels of E-cadherin and β-catenin in the piR-37524-inhibited groups. All statistical comparisons depicted in the figures were performed between the piR-37524 inhibitor-transfected group and the Inhibitor-NC group. Data are presented as the mean ± SD. *p < 0.05, **p < 0.01, ***p < 0.001, ****p < 0.0001 compared to the control group, Scale bar: 200 µm. n = 3

Given the upregulation of piR-37524 in CRC tissues and its correlation with tumor progression, highlighting its potential as a diagnostic biomarker, we investigated whether piR-37524 functions as an oncogene in colorectal cancer progression. We first evaluated piR-37524 expression across a panel of colorectal cancer cell lines and found that HCT116 and HT29 cells displayed markedly higher piR-37524 levels than the other lines (Fig. 5A). We then used an MTT assay to determine the proliferation rate of colorectal cancer cells transfected with piR-37524 inhibitors or NC. Our results showed that knockdown of piR-37524 significantly reduced the proliferation of HCT116 and HT29 cells (Fig. 5B,C). To further validate the effect of piR-37524 on cell proliferation, we performed a colony formation assay. In this assay, HCT116 and HT29 cells transfected with piR-37524 inhibitors were allowed to grow for a prolonged period, and the number of colonies formed was counted. The results revealed that knockdown of piR-37524 significantly reduced the colony-forming ability of both HCT116 and HT29 cells (Fig. 5D,E), further confirming its inhibitory effect on cell proliferation. Additionally, we performed wound healing assays to assess the effect of piR-37524 knockdown on cell migration. The results indicated that knockdown of piR-37524 markedly inhibited the migration of HCT116 and HT29 cells compared to the control treatment (Fig. 5F,G). WB analysis revealed a notable reduction in Snail protein expression within the HCT116 cell line following piR-37524 inhibition, and elevated protein levels of E-cadherin and β-catenin were observed in both HCT116 and HT29 cell lines. These alterations are indicative of changes in the epithelial-mesenchymal transition (EMT) process (Fig. 5H). Collectively, these findings demonstrate that inhibition of piR-37524 significantly impairs migration-related phenotypes of colorectal cancer cells *in vitro*. Effects on invasive capacity were not assessed in the present study and will be addressed in future work.

### 3.5 TNFAIP3 Is a Potential Target Gene of piR-37524 in HCT116 and HT29 Cells

**Figure 6:**
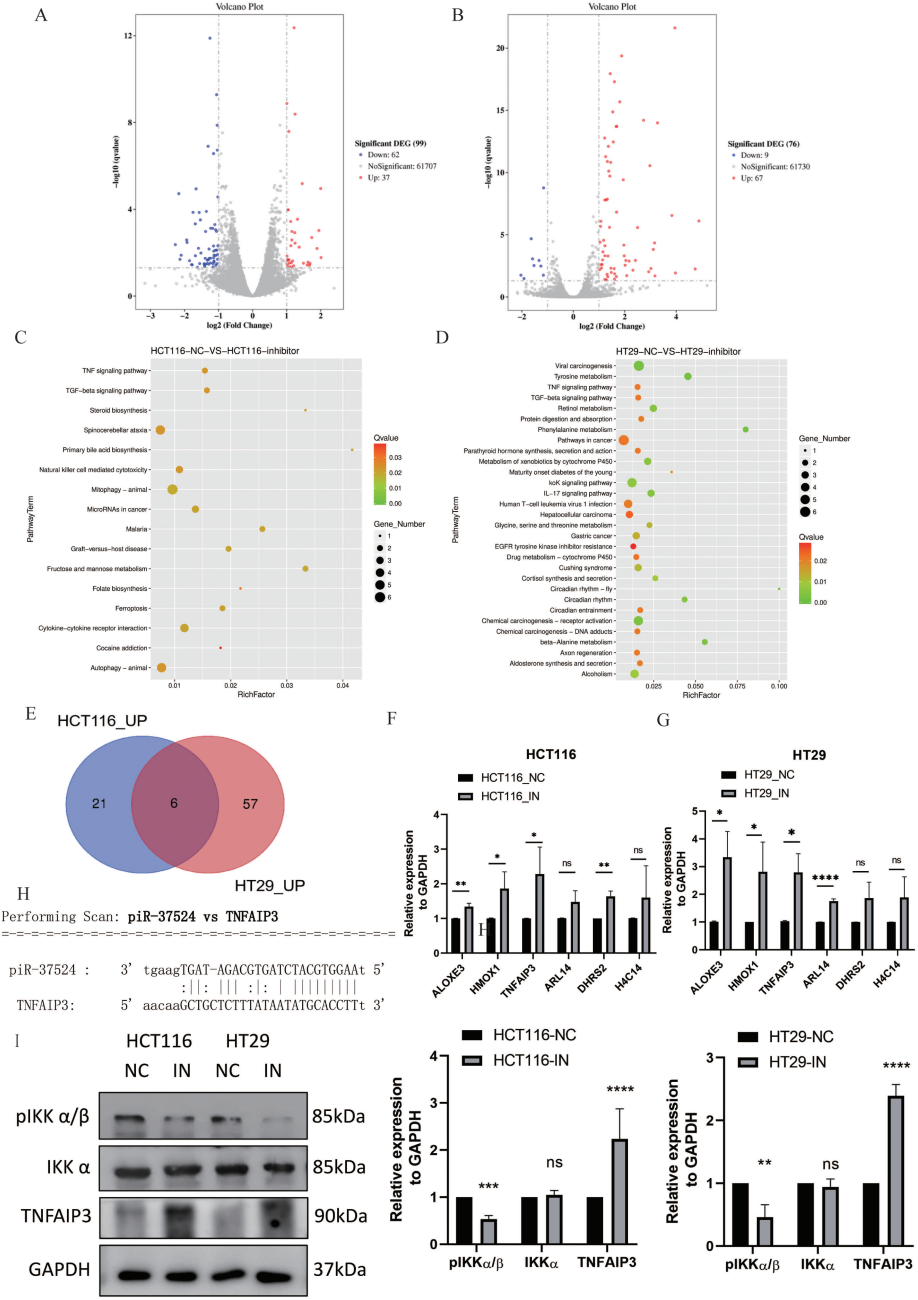
Identification and validation of DEGs upon piR-37524 inhibition in HCT116 and HT29 cells. Volcano plots showing DEGs in HCT116 (**A**) and HT29 (**B**) cells transfected with piR-37524 inhibitors compared to the negative control. Blue dots represent significantly downregulated genes, while red dots indicate significantly upregulated genes (*p* < 0.05, |log_2_ fold change| > 1). KEGG pathway enrichment analysis of DEGs in HCT116 (**C**) and HT29 (**D**) cells. The bubble plots show significantly enriched pathways, with the color gradient representing the q-value and the size of the bubbles indicating the number of genes. (**E**) Venn diagram showing the intersection of upregulated DEGs in HCT116 and HT29 cells, identifying six genes consistently upregulated in both cell lines. RT-qPCR validation of the expression of candidate DEGs in HCT116 (**F**) and HT29 (**G**) cells transfected with piR-37524 inhibitors compared to the negative control. Data are presented as mean ± SD (n = 3). *p < 0.05, **p < 0.01, ***p <0.001, ****p < 0.0001, ns: not significant. (**H**) Predicted interaction between piR-37524 and the 3^′^UTR of TNFAIP3 mRNA, identified using miRanda software. (**I**) Representative Western blots of E-cadherin, β-catenin, pIKK α/β, total IKK α, Snail, TNFAIP3, and GAPDH in HCT116 and HT29 cells transfected with piR-37524 inhibitors or NC

Accumulating evidence indicates that piRNAs can interact with mRNAs via partial sequence complementarity, thereby repressing or enhancing gene expression. To explore putative targets of piR-37524, we conducted RNA sequencing (GEO ID. GSE310036) on HCT116 and HT29 cells transfected with piR-37524 inhibitors or the corresponding negative control. In HCT116 cells, 83 differentially expressed genes (DEGs) were identified (*p* < 0.05, |log_2_ fold change| > 1), with 27 upregulated and 56 downregulated upon piR-37524 inhibition (Fig. 6A). In HT29 cells, 71 DEGs were identified, with 63 upregulated and 8 downregulated (Fig. 6B). KEGG enrichment analysis indicated that DEGs in both cell lines were associated with TNF and TGF-beta signaling pathways (Fig. 6C,D).

To further explore these potential target genes, we performed an intersection analysis of the upregulated DEGs in both HCT116 and HT29 cells and identified six genes consistently upregulated in both cell lines (Fig. 6E), of which ALOXE3, HMOX1, and TNFAIP3 were validated as significantly differentially expressed by RT-qPCR (Fig. 6F,G). Using miRanda software, we further analyzed and identified TNFAIP3 as a potential target gene of piR-37524. Our analysis suggested that piR-37524 might interact with the 3^′^UTRs of TNFAIP3 mRNA, which was upregulated by piR-37524 inhibitors (Fig. 6H). Thus, TNFAIP3 was identified as a candidate gene with a predicted 3^′^UTR binding site for piR-37524, supported by consistent upregulation at transcriptional and translational levels upon piR-37524 inhibition.

### 3.6 piR-37524 Regulates NF-**κ**B Signaling to Influence CRC Cell Migration

To further investigate the mechanism underlying migration inhibition, we evaluated the NF-κB signaling pathway in HCT116 and HT29 cells. Given that our previous RNA sequencing data suggested TNFAIP3 as a potential target of piR-37524, and TNFAIP3 is a known regulator of NF-κB signaling. First, TNFAIP3 expression was markedly increased in both HCT116 and HT29 cells following piR-37524 inhibition; then, we also examined the phosphorylation status of IKK α/β, a key component of the NF-κB pathway. Upon piR-37524 knockdown, we observed a decrease in the expression of phosphorylated IKK α/β (pIKK α/β), while the expression of total IKK α remained relatively unchanged (Fig. 6I). These findings suggest that piR-37524 may promote CRC cell migration and EMT by modulating the NF-κB signaling pathway, likely through a regulatory association with TNFAIP3.

## 4 Discussion

Nearly 2 decades back, PIWI protein-associated RNAs or piRNAs were identified as a novel class of small non-coding RNAs, primarily recognized for their role in germline development [17,18]. Over time, accumulating evidence has demonstrated that piRNAs extend their functions beyond transposon silencing to regulate gene expression in somatic tissues, including cancers [9,19–21]. Recently, our group published the earliest systematic NGS-based profiling of the CRC piRNAome and reported piR-24000 as a novel prognostic tissue-based biomarker in CRC that was found to be closely associated with an aggressive clinical presentation of the disease [8]. To expand our understanding of the piRNA biology associated with CRC, we revisited our NGS metadata to identify potential piRNA candidates differentially expressed in CRC. Through an organized filtering approach, we identified piR-37524 as a novel piRNA in CRC that was observed to be significantly overexpressed in CRC tissues relative to the tumor-adjacent non-cancerous tissues.

PiR-37524 (Accession: DQ599458; Aliases: piR-hsa-29715, PIR60569) is a 29-nucleotide small ncRNA located in chromosome 13 that has been previously reported to have an increased expression in a few cancers (Table S2). A global piRNA expression study undertaken using microarrays containing probes for 23,677 piRNAs to investigate differentially expressed piRNAs in bladder cancer identified over 5-fold upregulation of piR-37524 in bladder cancer tissues relative to the adjacent non-cancerous tissues [22]. Similar results were obtained by Zou et al. in head and neck squamous cell carcinoma (HNSCC) [23]. Specifically, this group profiled the RNA sequencing data from 422 HNSCC patients in The Cancer Genome Atlas (TCGA) and observed an increased expression of piR-hsa-29715 in patients with HNSCC.

In line with the literature, we confirmed that piR-37524 is upregulated in CRC and further demonstrated that higher piR-37524 expression is associated with larger tumour size, poor differentiation and the presence of distant metastases. Moreover, our study provided evidence demonstrating a significantly increased expression of the piRNA in adenomas as well as distant metastases (in the liver), suggesting a potential role of piR-37524 in the early stages of cancer development. ROC curves further demonstrated a potential role of the piRNA as a non-invasive, blood-based predictive diagnostic biomarker for the early identification of adenomas, which are primary risk factors for the development of CRC. While further investigations related to the actual molecular roles of the piRNA are warranted, a potential involvement of piR-37524 in the pre-cancerous stage (adenomatous polyps) or early cancer developmental stage at new sites (distant metastases) increases the likelihood of the molecule as a viable screening biomarker for cancer development or metastasis. Certainly, early identification of CRC has been shown to improve the overall patient survival significantly [24,25]. Recently, several piRNA molecules have been identified as potential biomarkers for early diagnosis of several cancers, including piR-651 and piR-823 in gastric cancer [26], piR-5937 and piR-28876 in colon cancer [15], piR-54265 for early detection and clinical monitoring of CRC [27], and piR-34536 and piR-5810 in clear cell renal cell carcinoma [28]. PiR-37524 was shown as a potential diagnostic biomarker in CRC in this study. It is warranted to investigate whether its performance can be further improved for clinical usage by combining it with other validated CRC biomarkers to develop a screening panel in CRC.

Beyond confirming its upregulation in tumor tissues and circulation, we sought to investigate the functional role and downstream effectors of piR-37524 on CRC cell lines. Knockdown of piR-37524 in HCT116 and HT29 cells significantly inhibited cell proliferation and migration, suggesting that piR-37524 acts as an oncogene in CRC. These *in vitro* findings corroborate our clinical observations of its association with tumor growth and metastasis. Emerging data indicate that piRNAs are able to interact with mRNAs via imperfect base pairing, thereby modulating gene expression through either repression or activation. For instance, Rakhmetullina et al. demonstrated piRNA-mRNA interactions in esophageal squamous cell carcinoma, highlighting this binding mode as a key regulatory mechanism [29]. Xie et al. found piRNA-14633 regulates cervical cancer cell malignancy by targeting mRNA in an m6A-dependent manner [30], while Ou et al. showed exosomal piRNA-17560 modulates breast cancer EMT via mRNA binding [31]. Beyond solid tumors, Ben et al. and Peng et al. further confirmed piRNAs’ ability to regulate mRNA translation or expression in prostate cancer, supporting the broad relevance of this regulatory pattern [32,33]. To identify potential targets of piR-37524, we performed RNA sequencing upon piR-37524 inhibition. RNA sequencing identified differentially expressed genes associated with the TNF signaling pathways, with TNFAIP3 emerging as a potential target gene. TNFAIP3, also known as A20, is a key negative regulator of the NF-κB signaling pathway and has been reported to have complex roles in cancer, with evidence suggesting both tumor-suppressing [34–36], and tumor-promoting activities [37] depending on the cancer type. In CRC, some studies suggest that TNFAIP3 may act as a tumor suppressor [38–40]. Our finding that piR-37524 inhibition leads to TNFAIP3 upregulation suggests a potential mechanism by which piR-37524 might promote CRC progression by suppressing the expression of this negative regulator. Notably, the predicted binding site between piR-37524 and the TNFAIP3 3^′^UTR suggests a direct post-transcriptional regulatory mechanism. Further investigation is needed to fully understand the intricate interplay between piR-37524 and TNFAIP3 in the context of CRC.

Furthermore, our investigation into the mechanism of migration inhibition revealed that piR-37524 knockdown led to the upregulation of epithelial markers (E-cadherin and β-catenin) and downregulation of the mesenchymal marker Snail, indicating an inhibition of the EMT [41,42]. EMT is a crucial process in cancer metastasis, and our findings suggest that piR-37524 may promote CRC metastasis by facilitating EMT. This observation aligns with the clinical correlation we found between high piR-37524 expression and distant metastasis. These results strongly suggest that piR-37524 promotes EMT and migration in CRC, at least partially, by suppressing TNFAIP3 and activating NF-κB-mediated Snail expression. Consistent with this, KEGG pathway enrichment from RNA-seq highlighted significant involvement of the TNF signaling pathway, which is intimately connected to NF-κB-driven EMT in CRC. Determining whether piR-37524 influences invasion and metastatic dissemination will require dedicated invasion assays and *in vivo* studies.

The observed decrease in pIKK α/β phosphorylation further supports the role of piR-37524 in activating the NF-κB pathway. IKK phosphorylation is a critical step in NF-κB activation, driving the degradation of IκB and nuclear translocation of p65/p50 complexes [43,44]. Our findings align with TNFAIP3’s known function as a negative regulator of IKK phosphorylation [45]. By upregulating TNFAIP3, piR-37524 inhibition likely enhances TNFAIP3-mediated deubiquitination of IKK complexes, reducing their phosphorylation and subsequent NF-κB activation. The reduction in pIKK α/β mirrors the downregulation of Snail and upregulation of E-cadherin observed in EMT assays. This suggests that piR-37524 promotes EMT by phosphorylating IKK and activating NF-κB, which transcriptionally upregulates Snail [46]. The lack of change in total IKK α further indicates specificity to phosphorylation status rather than protein abundance.

In conclusion, our study provides strong evidence for the overexpression of piR-37524 in CRC and its association with aggressive clinicopathological features. We have demonstrated its potential as a novel diagnostic and prognostic biomarker in CRC and have identified its oncogenic role *in vitro* by promoting cell proliferation and migration, potentially through the regulation of TNFAIP3/NF-κB/EMT axis. One limitation is that the direct binding between piR-37524 and TNFAIP3 mRNA remains unconfirmed; future investigations will utilize luciferase reporter assays and other targeted techniques to clarify this interaction. Additionally, *in vivo* models are required to validate the functional relevance of piR-37524 in CRC progression, and further studies should explore combinatorial therapeutic approaches involving piR-37524 inhibition to improve treatment outcomes. Importantly, combining piR-37524 with other piRNAs or protein-coding biomarkers may enhance diagnostic accuracy for early CRC and pre-cancerous lesions like adenomas, thereby contributing to improved patient outcomes.

## Supplementary Materials



## Data Availability

Data will be made available from the corresponding author upon reasonable request.

## Supplementary Materials

The supplementary material is available online at https://www.techscience.com/doi/10.32604/or.2025.074981/s1. References [47–56] are cited in the supplementary materials.
