# Supplementary material for: piR-37524 Overexpression in Colorectal Cancer: A Potential Diagnostic Bio-Marker and Therapeutic Target
Source: Oncol Res. 2026 Mar 23;34(4):34. doi: 10.32604/or.2026.074981 (PMC13040330; doi:10.32604/or.2026.074981)
Supplement: Supplementary file 1 [file OncolRes-34-74981-s001.docx]

**Supplementary Table S1**: Primer sequences used for reverse transcription and real-time PCR.

| **Gene Name** | **Direction** | **Primer Sequence (5′-3′)** |
| --- | --- | --- |
| Oligo dT for reverse transcription | - | CAGGTCCAGTTTTTTTTTTTTTTTTVN |
| piR-37524 | Forward | GCAGTAAGGTGCATCTAGTGCA |
|  | Reverse | GGTCCAGTTTTTTTTTTTTTTTACTTCACTAT |
| piR-31143 | Forward | AGCGTTGGTGGTATAGTGGTGA |
|  | Reverse | CAGTTTTTTTTTTTTTTTGCAGCTATGC |
| piR-39614 | Forward | GCAGTAGGATGTCGTGATGGGA |
|  | Reverse | CCAGTTTTTTTTTTTTTTTGAGCTCGG |
| piR-49145 | Forward | CAGTGAGGTAGTAGGTTGTATGGTTT |
|  | Reverse | GGTCCAGTTTTTTTTTTTTTTTGTAACTCT |
| piR-42111 | Forward | AGTCCAAAGGAGTGCTTGTGGA |
|  | Reverse | GTCCAGTTTTTTTTTTTTTTTGATCCGAT |
| piR-35406 | Forward | GCCCCTGTGATGAGTTGCCA |
|  | Reverse | CCAGTTTTTTTTTTTTTTTCCGTATTAGCA |
| RNU6B | Forward | CGCTTCGGCAGCACATATACTA |
|  | Reverse | ACGCTTCACGAATTTGCGT |
| hsa-miR-92a-3p | Forward | GCAGTATTGCACTTGTCCCG |
|  | Reverse | CAGGTCCAGTTTTTTTTTTTTTTTACAGGC |
| ALOXE3 | Forward | GCTGCTCTTCAATGCCATCCCT |
|  | Reverse | TGTCGTGAAGGTCTTATGGCACC |
| HMOX1 | Forward | CCAGGCAGAGAATGCTGAGTTC |
|  | Reverse | AAGACTGGGCTCTCCTTGTTGC |
| TNFAIP3 | Forward | CTCAACTGGTGTCGAGAAGTCC |
|  | Reverse | TTCCTTGAGCGTGCTGAACAGC |
| ARL14 | Forward | ACAGTCTGGGATGTTGGAGGAC |
|  | Reverse | CTGTCTCTGAGACTCTTCCAGTC |
| DHRS2 | Forward | GGTGCTGTCATCCTGGTCTCTT |
|  | Reverse | CCAGCTCCAATGCCAGTGTTCT |
| H4C14 | Forward | ACATTCAGGGCATCACCAAGCC |
|  | Reverse | TCTCCAGGAACACCTTCAGCAC |
| GAPDH | Forward | GTCTCCTCTGACTTCAACAGCG |
|  | Reverse | ACCACCCTGTTGCTGTAGCCAA |

**Supplementary Table S2**: Literature report of the piRNAs selected for preliminary investigation.

| **piRNA** | **Accession** | **Alias** | **Significantly Expressed piRNAs in CRC** | | **Role in Cancer** | **Reported Expression** |
| --- | --- | --- | --- | --- | --- | --- |
|  |  |  | **Fold Change** | ***p*-Value** |  |  |
| piR-37524 | DQ599458 | piR-hsa-29715, PIR60569 | 3.64 | 1.78 × 10^−3^ | Bladder cancer [22] | Upregulation |
|  |  |  |  |  | Head and neck squamous cell carcinoma [23] | Upregulation |
| piR-31143 | DQ571031 | piR-hsa-1282, PIR32142 | 4.32 | 1.96 × 10^−3^ | Multiple Myeloma [47] | Upregulation |
|  |  |  |  |  | Renal cell carcinoma [48,49] | Downregulation |
|  |  |  |  |  | Prostate carcinoma [50] | Upregulation |
|  |  |  |  |  | Gastric cancer [26, 51] | Downregulation |
|  |  |  |  |  | Breast cancer [50, 52] | Upregulation |
|  |  |  |  |  | Hepatocellular carcinoma [53] | Upregulation |
|  |  |  |  |  | Colorectal cancer [54, 55] | Upregulation |
| piR-39614 | DQ601548 | piR-hsa-31788, PIR62659 | −10.51 | 1.88 × 10^−2^ | Bladder cancer [22] | Upregulation |
| piR-49145 | DQ581033 | piR-hsa-11362, PIR42144 | −7.57 | 3.59 × 10^−7^ | Head and neck squamous cell carcinoma [23, 56] | Downregulation |
| piR-42111 | DQ573999 | piR-hsa-4276, PIR35110 | −6.61 | 1.66 × 10^−2^ | Not reported |  |
| piR-35406 | DQ597340 | piR-hsa-27615, PIR58451 | −3.86 | 3.16 × 10^−3^ | Not reported |  |
